# Matrix Metalloproteinase-9-Generated COOH-, but Not NH_2_-Terminal Fragments of Serum Amyloid A1 Retain Potentiating Activity in Neutrophil Migration to CXCL8, With Loss of Direct Chemotactic and Cytokine-Inducing Capacity

**DOI:** 10.3389/fimmu.2018.01081

**Published:** 2018-06-04

**Authors:** Mieke Gouwy, Mieke De Buck, Sara Abouelasrar Salama, Jennifer Vandooren, Sofie Knoops, Noëmie Pörtner, Lotte Vanbrabant, Nele Berghmans, Ghislain Opdenakker, Paul Proost, Jo Van Damme, Sofie Struyf

**Affiliations:** ^1^Laboratory of Molecular Immunology, Department of Microbiology and Immunology, Rega Institute for Medical Research, University of Leuven, Leuven, Belgium; ^2^Laboratory of Immunobiology, Department of Microbiology and Immunology, Rega Institute for Medical Research, University of Leuven, Leuven, Belgium

**Keywords:** serum amyloid A, chemokines, chemotaxis, induction, gelatinase B/matrix metalloproteinase-9

## Abstract

Serum amyloid A1 (SAA1) is a prototypic acute phase protein, induced to extremely high levels by physical insults, including inflammation and infection. Human SAA and its NH_2_-terminal part have been studied extensively in the context of amyloidosis. By contrast, little is known about COOH-terminal fragments of SAA. Intact SAA1 chemoattracts leukocytes *via* the G protein-coupled receptor formyl peptide receptor like 1/formyl peptide receptor 2 (FPR2). In addition to direct leukocyte activation, SAA1 induces chemokine production by signaling through toll-like receptor 2. We recently discovered that these induced chemokines synergize with intact SAA1 to chemoattract leukocytes *in vitro* and *in vivo*. Gelatinase B or matrix metalloproteinase-9 (MMP-9) is also induced by SAA1 during infection and inflammation and processes many substrates in the immune system. We demonstrate here that MMP-9 rapidly cleaves SAA1 at a known consensus sequence that is also present in gelatins. Processing of SAA1 by MMP-9 at an accessible loop between two alpha helices yielded predominantly three COOH-terminal fragments: SAA1(52–104), SAA1(57–104), and SAA1(58–104), with a relative molecular mass of 5,884.4, 5,327.3, and 5,256.3, respectively. To investigate the effect of proteolytic processing on the biological activity of SAA1, we chemically synthesized the COOH-terminal SAA fragments SAA1(52–104) and SAA1(58–104) and the complementary NH_2_-terminal peptide SAA1(1–51). In contrast to intact SAA1, the synthesized SAA1 peptides did not induce interleukin-8/CXCL8 in monocytes or fibroblasts. Moreover, these fragments possessed no direct chemotactic activity for neutrophils, as observed for intact SAA1. However, comparable to intact SAA1, SAA1(58–104) cooperated with CXCL8 in neutrophil activation and migration, whereas SAA1(1–51) lacked this potentiating activity. This cooperative interaction between the COOH-terminal SAA1 fragment and CXCL8 in neutrophil chemotaxis was mediated by FPR2. Hence, proteolytic cleavage of SAA1 by MMP-9 fine tunes the inflammatory capacity of this acute phase protein in that only the synergistic interactions with chemokines remain to prolong the duration of inflammation.

## Introduction

Serum amyloid A (SAA) is an acute phase protein, mainly produced in the liver under inflammatory conditions (1), but extrahepatic production of SAA has also been reported (2, 3). Moreover, SAA is involved in many inflammatory diseases, such as rheumatoid arthritis, diabetes type 2 and cancer (4–7). Several biological activities have been ascribed to SAA. At high concentrations (1–20 µg/ml), SAA has antiviral (8, 9) and antibacterial (10–12) activities and plays a role in cholesterol transport (13). More relevantly, at low concentrations (10–500 ng/ml), SAA induces cytokines, chemokines, and matrix metalloproteinases (MMPs) (14–17). One of the most important biological activities of SAA, exerted at similarly low concentrations, is its direct and indirect chemotactic activity for monocytes, neutrophils, immature dendritic cells, and regulatory T cells (17–21). On one hand, the direct chemotactic activity of SAA is mediated by the G protein-coupled receptor (GPCR) formyl peptide receptor 2 (FPR2) (22). On the other hand, SAA is indirectly chemotactic for leukocytes *via* induction of chemokines through binding to toll-like receptor 2 (TLR2) (17, 21). However, other receptors are also involved in cytokine induction by SAA variants (23–25). Following induction by serum amyloid A1 (SAA1), these chemokines synergize with each other, but also with SAA, to enhance leukocyte migration to the inflammatory site (17). Chemokines are small chemotactic cytokines, directing leukocyte migration under homeostatic and inflammatory conditions through binding to GPCRs (26). The chemokine family mainly consists of CC and CXC chemokines, depending on the position of the first two cysteine residues in their primary structure (27). Posttranslational truncation of chemokines can increase, decrease, or even completely block their chemotactic capacity (27, 28).

Several variants of SAA exist: SAA1, SAA2, SAA3, and SAA4. SAA1 and SAA2 are highly induced during the acute phase response (100- to 1,000-fold increase), whereas SAA4 is constitutively present in plasma at lower concentrations (3). Furthermore, truncated forms of circulating SAA have been detected in several diseases (29, 30). Proteolytic cleavage of SAA occurs through interaction with mostly MMPs (i.e., MMP-1, -2, and -3) (31, 32), but SAA can also be cleaved by other proteases (33). Nonetheless, the role of the different SAA variants and their processed forms in the pathogenesis of diseases and their potential distinct functions still need to be elucidated. We recently demonstrated that posttranslational cleavage of SAA variants by proteases may lead to alterations in their biological activities. Indeed, SAA1(47–104) cooperates with chemokines in neutrophil and monocyte migration and desensitizes the synergy between intact SAA1 and CXCL8 in neutrophil chemotaxis, suggesting that this peptide binds FPR2 (34). As its name suggests, SAA has been studied in inflammation-associated amyloidosis. However, being an acute phase reactant, SAA1 is present in the circulation and in exudates during infection or inflammation, even when no amyloidosis occurs. Matrix metalloproteinase-9 (MMP-9) or gelatinase B is a tuner and amplifier of immune functions (35), and its levels are increased in all inflammatory diseases (36).

To investigate whether MMP-9 can cleave SAA1, we incubated intact recombinant SAA1 with activated MMP-9 and identified the cleavage products *via* mass spectrometry. Three COOH-terminal SAA fragments were detected: SAA1(52–104), SAA1(57–104), and SAA1(58–104). Next, we chemically synthesized the most abundantly present SAA fragments, SAA1(52–104) and SAA1(58–104) and, in addition, the complementary NH_2_-terminal peptide SAA1(1–51), to determine their biological activity. In contrast to intact SAA1, the synthesized SAA1 peptides did not induce CXCL8 in monocytes, nor in fibroblasts. Moreover, SAA1(1–51), SAA1(52–104), and SAA1(58–104) did not chemoattract neutrophils. However, like intact SAA1, SAA1(58–104) still cooperated with CXCL8 in neutrophil activation and migration, through binding to FPR2, whereas SAA1(1–51) failed to exert this potentiating activity. These data show that proteolytic cleavage of SAA1 by MMP-9 reduces its chemotactic and chemokine-inducing capacity but not its cooperative potential, thereby fine-tuning the inflammatory response.

## Materials and Methods

### Reagents

Full length human proMMP-9/gelatinase B was expressed in Sf9 insect cells and purified to homogeneity by gelatin-Sepharose chromatography. ProMMP-9 was activated with the catalytic domain of MMP-3 (Merck Millipore, Darmstadt, Germany) as described (37). Afterward, MMP-9 activity was confirmed by SDS-PAGE of cleaved substrate and a gelatin degradation assay as previously described (38). Human (hu) recombinant intact apo-SAA1 (rSAA1α) containing an NH_2_-terminal methionine (11,814 kDa), CXCL8(6–77), and recombinant IL-1β were purchased from Peprotech (Rocky Hill, NJ, USA). Lipopolysaccharide (LPS) from *Escherichia coli* (0111:B4) was obtained from Sigma-Aldrich (St. Louis, MO, USA). The selective FPR2 antagonist WRW_4_ and the FPR2 agonist WKYMVm were purchased from Calbiochem (San Diego, CA, USA) and Phoenix Pharmaceuticals (Burlingame, CA, USA), respectively.

### Cleavage of Recombinant SAA1 With Gelatinase B/MMP-9

rSAA1α (7.5 µM) was incubated with activated human MMP-9 (0.15 µM) in assay buffer (100 mM Tris/HCl, pH 7.4, 100 mM NaCl, 10 mM CaCl_2_, 0.01% Tween-20) at 37°C for 0, 0.25, 0.5, 1, 2, 4, 8, 22, or 30 h. Control experiments were conducted under identical conditions without MMP-9. SAA1α cleavage products were detected by SDS-PAGE and Coomassie brilliant blue protein staining. In another set of experiments, mass spectrometric analysis of the SAA1α cleavage products was performed. Therefore, peptides derived from a 3 h incubation of SAA1α with MMP-9 in assay buffer without Tween-20 were first desalted using C4 ZipTips (Merck, Overijse, Belgium). Subsequently, these peptides were diluted in 50% acetonitrile/50% H_2_O/0.1% acetic acid and peptide solutions were analyzed by electrospray mass spectrometry (MS) (Amazon-SL, Bruker Daltonics, Bremen, Germany).

### Chemical Synthesis of SAA1 Peptides

hu SAA1(1–51), hu SAA1(52–104), and hu SAA1(58–104) were chemically synthesized based on Fmoc [*N*-(9-fluorenyl)methoxycarbonyl] chemistry using an Activo-P11 automated solid-phase peptide synthesizer (Activotec, Cambridge, UK), deprotected and purified as described previously (39).

### Cell Cultures

CD14^+^ monocytes were isolated from human 1-day-old buffy coats, derived from healthy donors (Blood Transfusion Center of the Red Cross, Mechelen, Belgium), *via* density gradient centrifugation on Pancoll separating solution (density 1.077 g/ml; PAN Biotech, Aidenbach, Germany) and *via* positive selection (MACS, Miltenyi Biotec, Bergisch Gladbach, Germany) (40). Neutrophils were isolated from fresh blood, derived from healthy donors, *via* density gradient centrifugation as described (21). Human embryonic diploid skin-muscle fibroblasts (E_6_SM cells) were grown in minimal essential medium (MEM; Lonza, Verviers, Belgium) containing 10% FCS.

### Neutrophilic Granulocyte Activation Assays

Neutrophil migration was measured in the Boyden microchamber assay (Neuro Probe, Gaithersburg, MD, USA) as previously described (21). The chemotactic index (CI) was calculated by dividing the average number of cells migrated to the chemotactic factor by the average number of cells migrated to the chemotaxis control buffer. Synergy was obtained when the net chemotactic index (net CI = CI − 1) of the combined chemotactic substances was significantly higher than the sum of the net CI of the chemotactic substances added separately to the microchamber. For antagonizing experiments, the upper wells of the Boyden microchamber were loaded with neutrophils in the presence of the FPR2 antagonist WRW_4_ (20 µg/ml).

The shape change test was used to measure fast and direct activation of neutrophils in suspension. Purified human neutrophils (0.6 × 10^6^ cells/ml) were diluted in Hanks’ balanced salt solution (HBSS; Life Technologies, Paisley, UK) supplemented with 10 mM HEPES (Life Technologies) and incubated in a 96-well microtiter plate in the presence of dilution buffer, CXCL8, SAA1(58–104), or a combination of CXCL8 and SAA1(58–104). After 3 min of stimulation, the cells were fixed by adding an equal volume of HBSS/HEPES buffer containing 4% formaldehyde. For each condition, 100 cells, morphologically identified as round, blebbed, or elongated cells, were counted microscopically (magnification 200×) and independently by two individuals in a blinded manner. For the assessment of cooperation, the net percentages of neutrophils undergoing shape change (blebbed + elongated cells) were used.

### Chemokine Induction Experiments

Monolayers of primary human fibroblasts were grown to confluency in 48-well plates in MEM containing 10% FCS. Cells were stimulated in MEM containing 2% FCS for 24 h with different doses of IL-1β, SAA1α, the COOH-terminal, and NH_2_-terminal SAA1 peptides or were left untreated (control). CD14^+^ monocytes were seeded in 48-well plates in RPMI 1640 medium supplemented with 0.5% human serum albumin (Belgian Red Cross, Brussels, Belgium) at a concentration of 2 × 10^6^ cells/ml and induced for 24 h with different doses of LPS, SAA1α, SAA1(1–51), SAA1(52–104), and SAA1(58–104) or were left untreated (control). Levels of human CXCL8 were quantified by a specific sandwich ELISA developed in our laboratory (lowest level of detection: 0.04 ng/ml) (41).

### Statistical Analysis

Data were first analyzed by the non-parametric Kruskal–Wallis test (Statistica 12.0) for comparison of multiple groups before performing pairwise comparisons. The Mann–Whitney *U* test and the Wilcoxon Sum Rank test were used to compare data from two unpaired or paired data sets, respectively.

## Results

### Cleavage of SAA1 by Gelatinase B/MMP-9

**Figure 1:**
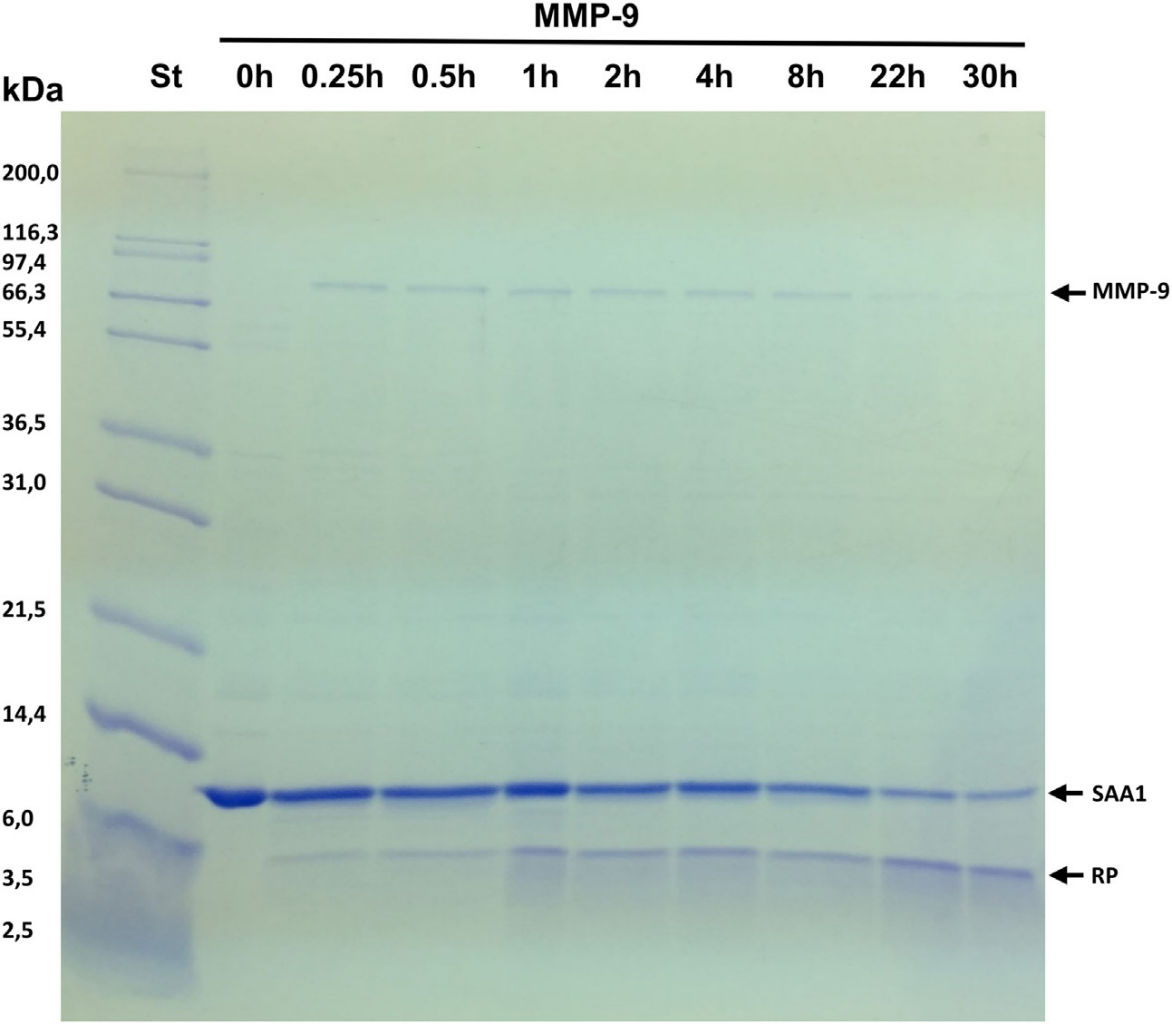
Cleavage of serum amyloid A1 (SAA1) by matrix metalloproteinase-9 (MMP-9). Pro-MMP-9 was activated with the catalytic domain of MMP-3, and recombinant human SAA1 was incubated at 37°C with activated gelatinase B/MMP-9 at an enzyme:substrate molar ratio of 1:50. Samples taken at the indicated time points were analyzed by SDS–PAGE and Coomassie brilliant blue protein staining. The relative molecular weight of the standard marker proteins (St) is indicated in kilodaltons. After incubation for prolonged time intervals (22 and 30 h), some autocatalysis of MMP-9 was detected, slowing down the conversion of SAA1 into reaction products (RP).

To determine whether SAA1 is cleaved by gelatinase B/MMP-9, recombinant intact SAA1 (11.8 kDa) was incubated at 37°C with the enzyme at a 1:50 enzyme:substrate ratio and analyzed by SDS–PAGE. Figure 1 shows a clear shift of the protein mass toward 5 kDa reaction products after incubation of SAA1 with MMP-9, demonstrating that MMP-9 processes the acute phase protein SAA1, presumably by cleaving it in the middle of the protein. The kinetics of the cleavage of SAA1 by MMP-9 were studied by taking samples at various time intervals during the incubation (Figure 1). Cleavage of SAA1 by MMP-9 resulted in a decrease of the intensity of intact SAA1 over time. The cleavage products were already visible after 0.25 h, and the intensity of the SAA1 cleavage products increased over time. However, MMP-9 treatment did not lead to complete degradation of intact SAA1 after 30 h.

**Figure 2:**
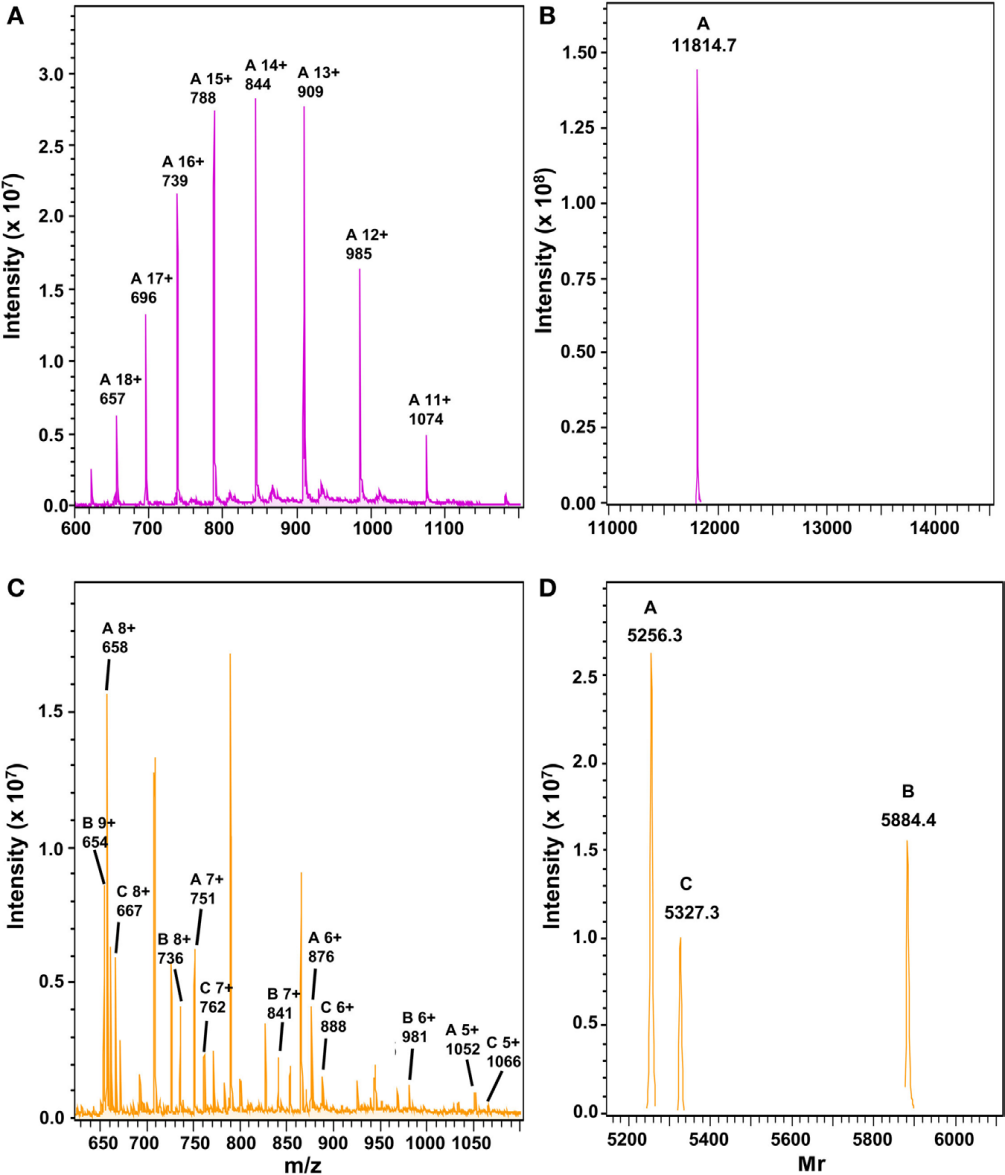
Mass spectrometric analysis of serum amyloid A1 (SAA1) after cleavage with matrix metalloproteinase-9 (MMP-9). Recombinant SAA1 was analyzed by electrospray ion trap mass spectrometry before **(A,B)** and after **(C,D)** incubation for 3 h at 37°C with active MMP-9. The unprocessed **(A,C)** and charge-deconvoluted **(B,D)** spectra are shown. The theoretical average relative molecular masses (*M*_r_) of intact SAA1, SAA1(52–104), SAA1(57–104), and SAA1(58–104) are 11,814.9, 5,884.4, 5,327.7, and 5,256.7, respectively.

To determine the exact cleavage sites of MMP-9 in SAA1, intact SAA1 was incubated with the enzyme and subsequently analyzed by MS (Figure 2). After 3 h, intact SAA1 (Figures 2A,B) was cleaved by MMP-9 into three different COOH-terminal fragments: SAA1(52–104), SAA1(57–104), and SAA1(58–104) (Figures 2C,D). The relative intensities of the different peptide peaks are indicated in Table 1. The cleavage thus occurred after a glycine residue at position 51 and yielded a COOH-terminal part of similar size starting with valine at position 52. This cleavage site is within a small protein loop between two alpha helices of SAA1 (42) and coincides with the sequon Gly–Pro–Xaa–Gly–hydrophobic residue, which is the preferred consensus sequence and cleavage site of MMP-9 in denatured collagens or gelatins (43).

**Table 1:** Mass spectrometric analysis of the cleavage sites of matrix metalloproteinase-9 (MMP-9) in intact recombinant SAA1α.

a.a.	Intact serum amyloid A1 (SAA1) protein sequence	
1–104^b^	RSFFSFLGEAFDGARDMWRAYSDMREANYIGS DKYFHARGNYDAAKRGPGG VWAAEAISDARENIQRFFGHGAEDSLADQAANEWGRSGKDPNHFRPAGLPEKY	

	**COOH-terminal SAA1 peptide sequence**	**Rel. int. (%),^a^ 3 h**

52–104	VWAAEAISDARENIQRFFGHGAEDSLADQAANEWGRSGKDPNHFRPAGLPEKY	29.9
57–104	AISDARENIQRFFGHGAEDSLADQAANEWGRSGKDPNHFRPAGLPEKY	19.8
58–104	ISDARENIQRFFGHGAEDSLADQAANEWGRSGKDPNHFRPAGLPEKY	50.3

*^a^Relative intensity (%) of the SAA1(52–104), SAA1(57–104), and SAA1(58–104) peptides after incubation for 3 h of intact SAA1α (Uniprot P0DJI8) with MMP-9 (E:S ratio 1:50) and subsequent analysis by mass spectrometry*.

*^b^The amino acid (a.a.) sequence of SAA1(1–51) is underlined in the intact protein sequence*.

### Chemical Synthesis and Purification of SAA1 Peptides

**Figure 3:**
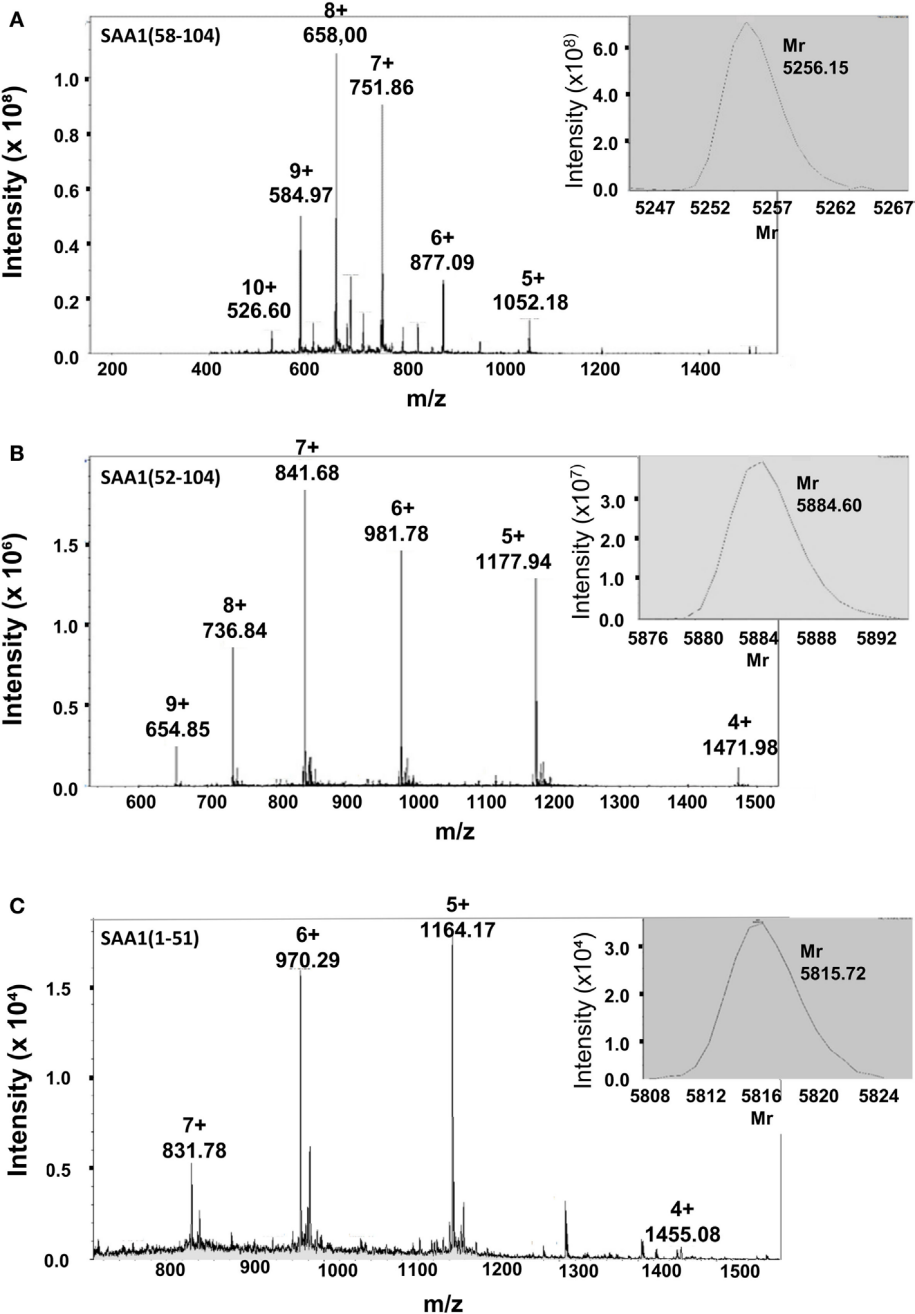
Relative molecular mass determination of chemically synthesized, HPLC-purified SAA1(1–51), SAA1(52–104), and SAA1(58–104) by mass spectrometric analysis. The COOH-terminal peptides SAA1(58–104) **(A)** and SAA1(52–104) **(B)** and the NH_2_-terminal peptide SAA1(1–51) **(C)** were chemically synthesized, deprotected, and purified *via* RP-HPLC. Fractions containing peptides with a correct *M*_r_ and with sufficient purity were pooled. The averaged mass spectra of these pools are shown with the ion intensities, the number of charges and the corresponding mass over charge ratio (*m*/*z*) for multiple charged ions. The deconvoluted experimentally determined mass spectra, as calculated by the Bruker deconvolution software, with the *M*_r_ of the uncharged serum amyloid A1 (SAA1) peptides are shown as inserts at the upper right of the averaged mass spectra.

Intact recombinant SAA1 chemoattracts neutrophils and potently synergizes with CXCL8 in neutrophil activation and chemotaxis *via* its GPCR FPR2 (21). To better define the role of the SAA1 fragments in inflammation, the predominant COOH-terminal fragments SAA1(52–104) and SAA1(58–104) and the complementary NH_2_-terminal SAA1 fragment SAA1(1–51) were synthesized by Fmoc chemistry on a solid-phase peptide synthesizer and purified to homogeneity by RP-HPLC. Ion trap mass spectrometric analysis confirmed the correct synthesis and deprotection of SAA1(58–104) [relative molecular mass (*M*_r_) of 5,256.2; Figure 3A], SAA1(52–104) (*M*_r_ of 5,884.6; Figure 3B), and SAA1(1–51) (*M*_r_ of 5,815.7; Figure 3C). After purification, SAA1(1–51), SAA1(52–104), and SAA1(58–104) peptides were used in various biological assays.

### SAA1 Peptides Fail to Induce CXCL8 in CD14^+^ Monocytes and Fibroblasts

**Figure 4:**
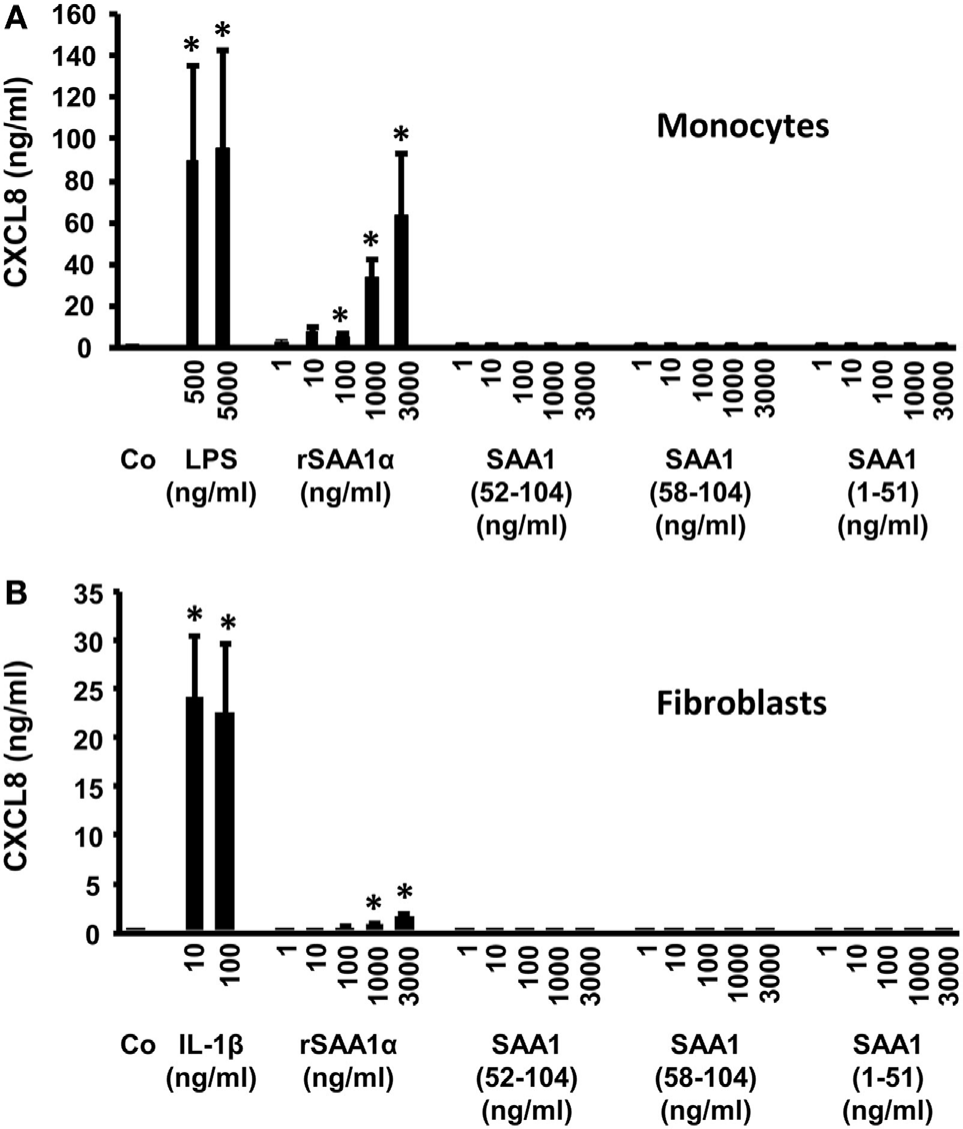
SAA1(1–51), SAA1(52–104), and SAA1(58–104) fail to induce CXCL8 in monocytes and fibroblasts. Human CD14^+^ monocytes **(A)** or diploid fibroblasts **(B)** were incubated with recombinant intact serum amyloid A1 (SAA1) (1–3,000 ng/ml), SAA1(1–51) (1–3,000 ng/ml), SAA1(52–104) (1–3,000 ng/ml), SAA1(58–104) (1–3,000 ng/ml), lipopolysaccharide (LPS) [500 or 5,000 ng/ml **(A)**], or IL-1β [10 or 100 ng/ml **(B)**] for 24 h. Results represent the mean ± SEM CXCL8 production from four independent experiments. Asterisks indicate significant induction of CXCL8 compared with untreated (Co) samples (Mann–Whitney *U* test; *p < 0.05).

Recently, we showed that SAA1 is a potent inducer of CXCL8 in CD14^+^ monocytes (17). To verify whether SAA1(1–51), SAA1(52–104), and SAA1(58–104) induced chemokines, we stimulated human CD14^+^ monocytes and diploid fibroblasts with different concentrations of the SAA1 peptides (1–3,000 ng/ml), and the amount of CXCL8 in the cell supernatants was measured by a specific ELISA (Figure 4). After stimulation for 24 h with SAA1 or LPS, CD14^+^ monocytes produced statistically significant amounts of CXCL8. Indeed, a maximal concentration of 63.1 ± 30.5 ng/ml (*n* = 4, *p* = 0.03) and 95.5 ± 46.7 ng/ml (*n* = 4, *p* = 0.03) of CXCL8 was produced upon stimulation of CD14^+^ monocytes with 3,000 ng/ml SAA1 or 5,000 ng/ml LPS, respectively (Figure 4A). However, no detectable induction of CXCL8 could be observed after 24 h in monocytes stimulated with different concentrations of SAA1(1–51), SAA1(52–104), and SAA1(58–104).

In fibroblasts, similar results were obtained. The COOH- and NH_2_-terminal SAA1 peptides failed to induce CXCL8, whereas stimulation with intact SAA1 (1,000 and 3,000 ng/ml) provoked a weak (0.6 ± 0.4 and 1.4 ± 0.4 ng/ml CXCL8, respectively), but statistically significant (*p* = 0.03) induction of CXCL8. IL-1β (10 or 100 ng/ml) was used as a positive control, and this cytokine was found to be a stronger CXCL8 inducer (24.0 ± 7.1 ng/ml at 10 ng/ml IL-1β) in fibroblasts (Figure 4B).

### Cleavage of SAA1 by MMP-9 Reduces Its Chemotactic Activity for Neutrophils

**Figure 5:**
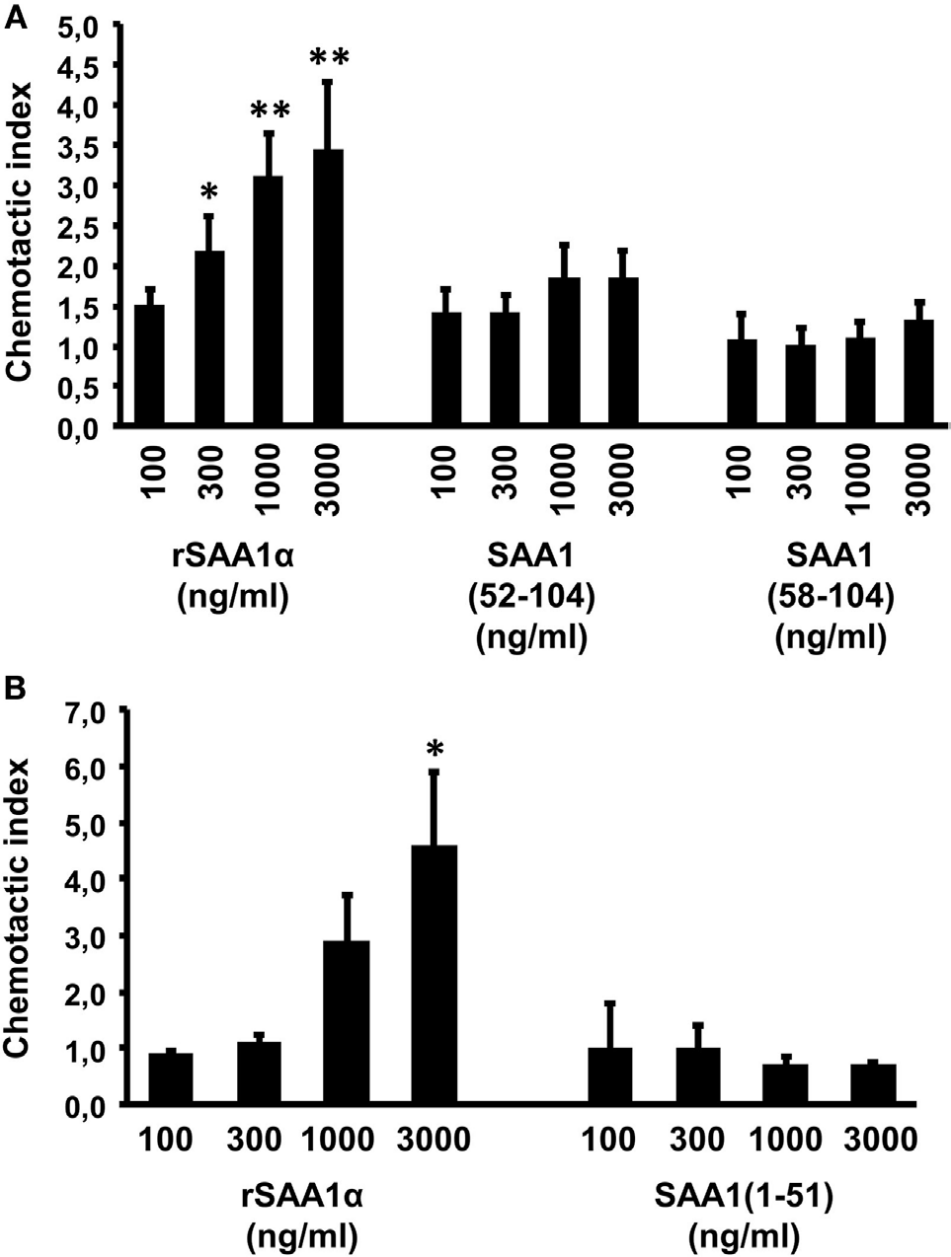
The NH_2_- and COOH-terminal serum amyloid A1 (SAA1) peptides are not directly chemotactic for neutrophils. The chemotactic activity of 100–3,000 ng/ml intact SAA1 **(A,B)**, SAA1(1–51) **(B)**, SAA1(52–104) **(A)**, and SAA1(58–104) **(A)** was evaluated on human neutrophils in the Boyden microchamber assay. The chemotactic potencies are expressed as mean chemotactic index ± SEM from four **(B)** to six **(A)** independent experiments. Statistically significant differences compared with controls, determined by the Mann–Whitney *U* test, are indicated by asterisks (*p < 0.05 and **p < 0.01).

Previous reports showed that enzymatic proteolysis is an important posttranslational regulatory mechanism to control chemokine activity (28, 44). Here, we tested the effect of SAA1 cleavage by MMP-9 on its capacity to stimulate neutrophil migration. Different concentrations of intact SAA1, SAA1(1–51), SAA1(52–104), and SAA1(58–104) were applied in the *in vitro* Boyden microchamber chemotaxis assay (Figure 5). The results indicated that, in contrast to intact SAA1, the COOH-terminal (Figure 5A) and NH_2_-terminal SAA1 peptides (Figure 5B) no longer induced significant neutrophil migration at concentrations as high as 3,000 ng/ml. By contrast, 1,000 ng/ml of intact SAA1 significantly stimulated the migration of neutrophils, reaching a CI of 3.1 ± 0.5 (*n* = 6) (Figure 5A) or 2.9 ± 0.8 (*n* = 4) (Figure 5B).

### SAA1(58–104), but Not SAA1(1–51), Enhances Neutrophil Chemotaxis in Response to CXCL8

**Figure 6:**
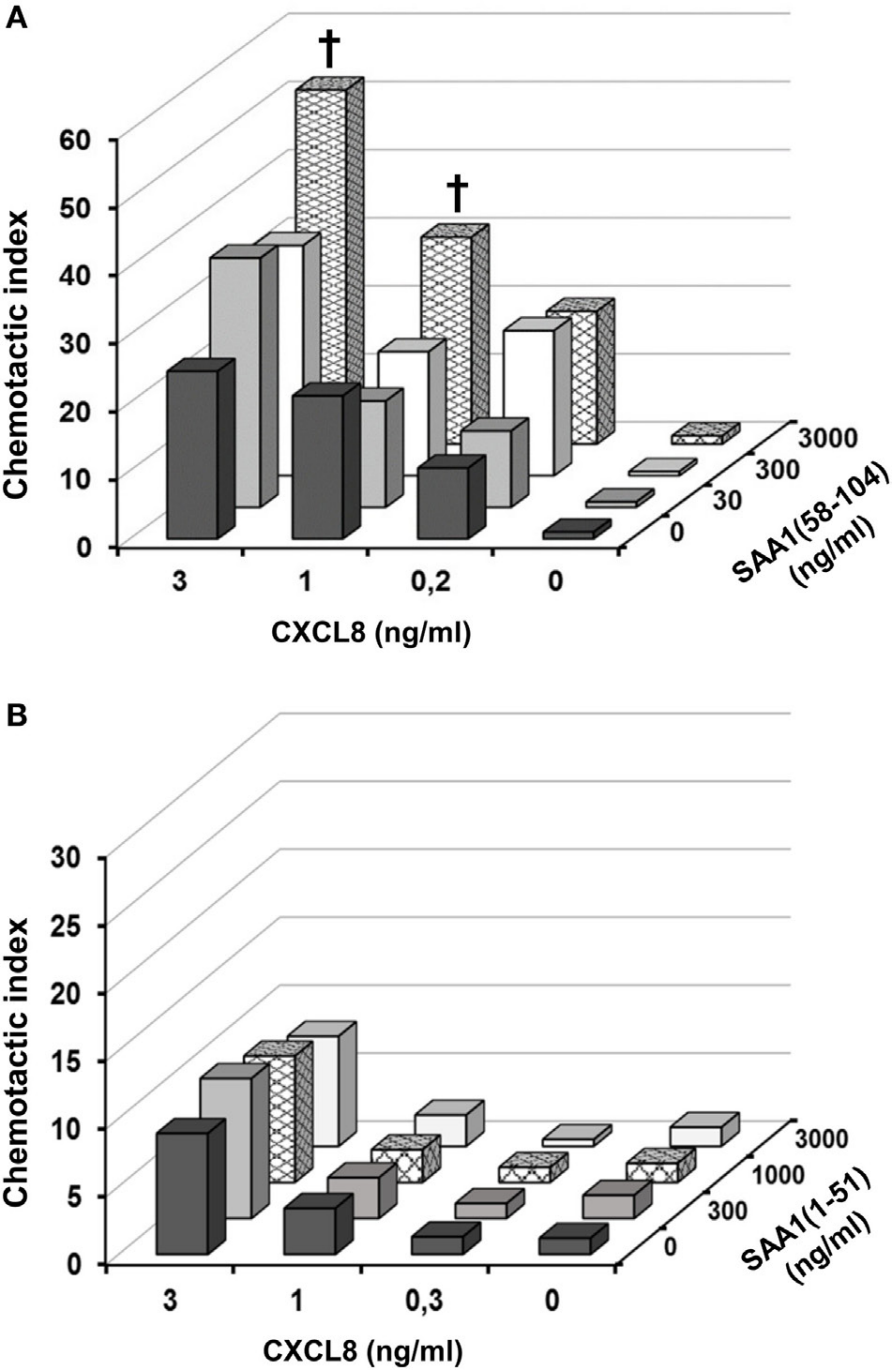
SAA1(58–104), but not SAA1(1–51), cooperates with CXCL8 in neutrophil chemotaxis. The COOH-terminal peptide SAA1(58–104) (30–3,000 ng/ml) **(A)** and the NH_2_-terminal peptide SAA1(1–51) (300–3,000 ng/ml) **(B)** were added in the presence or absence of CXCL8 (0.2–3 ng/ml) to the lower compartment of the Boyden microchamber to measure human neutrophil chemotaxis. The chemotactic response is expressed as the mean chemotactic index ± SEM derived from three to five independent experiments. Statistically significant cooperation, determined by the Mann–Whitney *U* test, is indicated by daggers (^†^*p* < 0.05).

We have previously shown that intact SAA1 synergizes with CXCL8 in neutrophil migration and activation (21). Therefore, we also evaluated the effect of SAA1 cleavage on the occurrence of its cooperation with CXCL8 in neutrophil activation and chemotaxis. CXCL8 (0.2–3 ng/ml), SAA1(58–104) (30–3,000 ng/ml), or SAA1(1–51) (300–3,000 ng/ml) were combined in the lower wells of the Boyden microchamber assay (Figure 6). Neutrophils migrated toward CXCL8 in a concentration-dependent manner, whereas SAA1(58–104) or SAA1(1–51) did again not chemoattract neutrophils on their own (over a range of concentrations) (Figures 6A,B). However, when combined with 1 or 3 ng/ml of CXCL8, SAA1(58–104) at 3,000 ng/ml significantly (*p* = 0.03 and *p* = 0.02, respectively) increased CXCL8-mediated neutrophil chemotaxis (Figure 6A). Similar observations were made with SAA1(52–104) (34). By contrast, the NH_2_-terminal SAA1(1–51) peptide did not cooperate with CXCL8 in neutrophil chemotaxis (Figure 6B).

**Figure 7:**
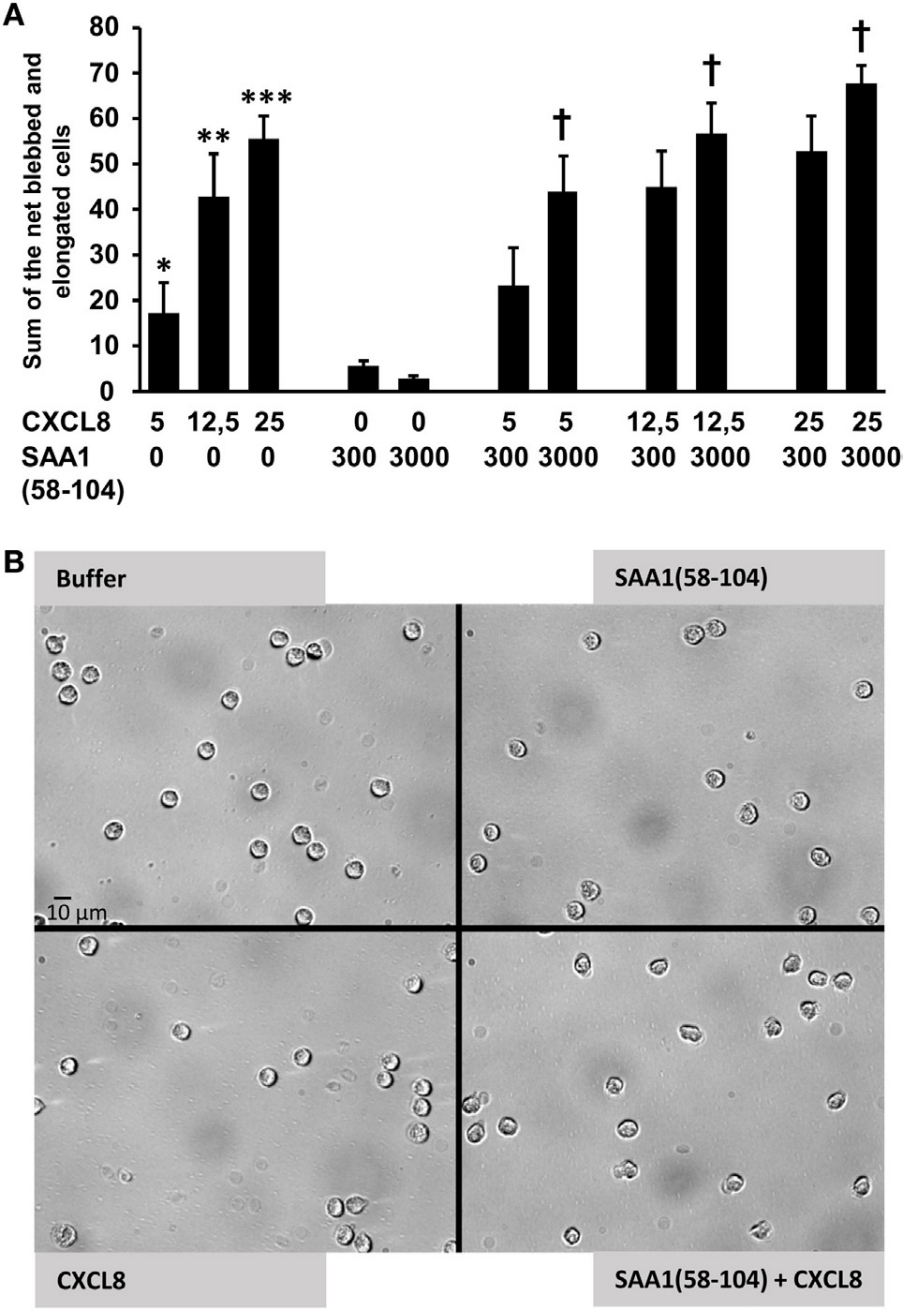
SAA1(58–104) cooperates with CXCL8 in neutrophil activation. **(A)** Neutrophils were treated with buffer, CXCL8 (5, 12.5, or 25 ng/ml), SAA1(58–104) (300 or 3,000 ng/ml), or a combination of CXCL8 and SAA1(58–104). Data from 8 to 16 independent experiments are expressed as the net percentage of blebbed plus elongated neutrophils ± SEM. Statistically significant differences compared with controls and statistically significant cooperation, determined by the Wilcoxon Sum Rank test, are indicated by asterisks (*p = 0.05; **p < 0.01; and ***p < 0.001) or by a dagger (^†^*p* < 0.05), respectively. **(B)** Bright field pictures (40× magnification; scale bar 10 µm) illustrating the shape change of neutrophils after stimulation with buffer, SAA1(58–104) (3,000 ng/ml), CXCL8 (5 ng/ml), or the combination of SAA1(58–104) (3,000 ng/ml) and CXCL8 (5 ng/ml).

In shape change assays, SAA1(58–104) and CXCL8 also cooperated to activate neutrophils (Figure 7). Neutrophils treated with buffer or SAA1(58–104) alone remained spherical in shape. For the combination of 3,000 ng/ml SAA1(58–104) and CXCL8 at 5, 12.5, and 25 ng/ml, the net percentage of blebbed and elongated neutrophils was significantly higher (44 ± 8, *p* = 0.028; 57 ± 6, *p* = 0.04 and 68 ± 4, *p* = 0.01, respectively) than the sum of the percentages for SAA1(58–104) (3 ± 1%) or CXCL8 at 5, 12.5, or 25 ng/ml (18 ± 7; 43 ± 9; 56 ± 5, respectively) when added separately to the cells. These data demonstrate that COOH-terminal cleavage products of SAA1 generated by MMP-9 retain their cooperative potency with CXCL8 in neutrophil activation and migration.

### The Cooperative Effect Between CXCL8 and the COOH-Terminal Peptides of SAA1 in Neutrophil Chemotaxis Is Inhibited by the Selective FPR2 Antagonist WRW_4_

**Figure 8:**
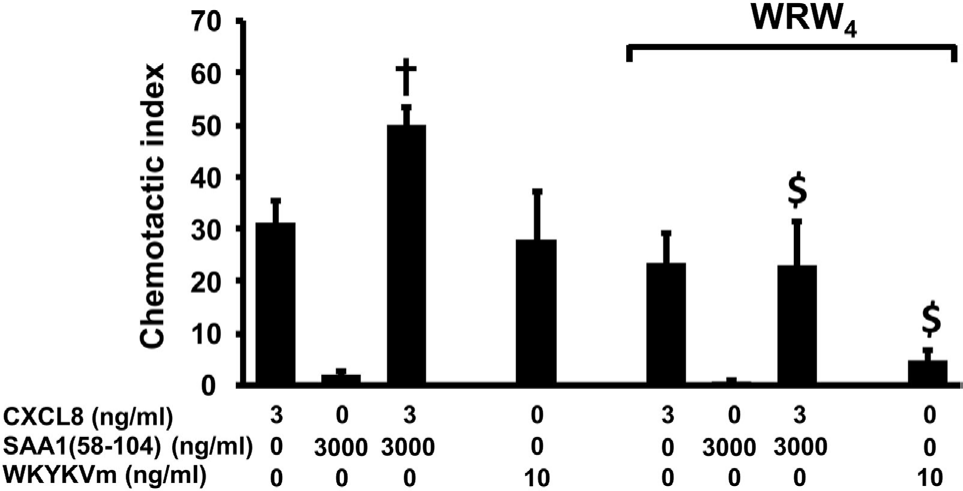
The selective formyl peptide receptor 2 antagonist WRW_4_ blocks the cooperation between SAA1(58–104) and CXCL8 in neutrophil chemotaxis. WKYMVm (10 ng/ml), CXCL8 (3 ng/ml), SAA1(58–104) (3,000 ng/ml), or a combination of CXCL8 and SAA1(58–104) were added to the lower compartment of the Boyden microchamber to measure neutrophil chemotaxis. WRW_4_ (20 µg/ml) or buffer was added to the cells just before loading the cells in the upper compartment of the microchamber. Data represent the mean chemotactic index (CI) ± SEM and are pooled from four to six independent experiments. Statistically significant cooperation is indicated by a dagger (compared with the sum of the net CI values when both chemotactic agents are tested separately; ^†^*p* < 0.05) and statistically significant inhibition by the antagonist is indicated by dollar signs (^$^*p* < 0.05; Mann–Whitney *U* test).

To investigate whether the cooperation between the COOH-terminal SAA1 peptides and CXCL8 in neutrophil chemotaxis implies the binding of SAA1(58–104) to the SAA1 receptor FPR2, the combination of SAA1(58–104) and CXCL8 was evaluated in the microchamber assay in the presence of the specific FPR2 antagonist WRW_4_ (Figure 8). In the absence of the FPR2 antagonist, SAA1(58–104) at 3,000 ng/ml (CI = 1.9 ± 0.7) cooperated (CI = 49.8 ± 3.6; *n* = 4; *p* = 0.03) with CXCL8 at 3 ng/ml (CI = 31.0 ± 4.6). Treatment of neutrophils with WRW_4_ at 20 µg/ml significantly (*p* = 0.03) reduced the observed potentiating effect (CI = 22.9 ± 8.3; *n* = 4) between SAA1(58–104) and CXCL8. As a control, the chemotactic effect of the FPR2 agonist WKYKVm (10 ng/ml; CI = 27.8 ± 9.6) was also significantly (*p* = 0.03) inhibited when neutrophils were treated with WRW_4_ (20 µg/ml) (CI = 4.6 ± 1.9; *n* = 4) (Figure 8). Similar FPR2-mediated cooperation in neutrophil migration was obtained for the combination of SAA1(52–104) and CXCL8 (data not shown).

## Discussion

Proteolytic processing of inflammatory mediators is a common feature to control the innate immune response. Indeed, inflammation is steered by proteases cleaving cytokines, chemokines, acute phase proteins, etc. Some cytokines such as IL-1 are naturally produced as precursor proteins which need to be enzymatically processed. The generated fully active endogenous pyrogen induces acute phase proteins, chemokines, and other cytokines in various cell types including leukocytes, fibroblasts, hepatocytes, and endothelial cells (45–47). Chemokines, which are not produced as pro-peptides, are confronted with many different immune cell-derived enzymes resulting in a complex chemokine–protease interacting network (28, 48). For example, soluble or membrane-associated CD26/DPP4 can NH_2_-terminally truncate most inflammatory chemokines with a different outcome on their biological effects. Indeed, the CXCR3 ligands lose their chemotactic activity upon removal of their two NH_2_-terminal residues, whereas such truncation renders some CC chemokines (e.g., CCL3) more active on specific leukocyte types (27, 28). Furthermore, chemokines such as CXCL8 stimulate neutrophils to secrete proteases such as gelatinase B/MMP-9, which in turn cleaves CXCL8 into a more active neutrophil chemoattractant (49). As a consequence, proteolytic processing of chemokines can enhance or dampen the inflammatory response (28). Similarly, the acute phase protein SAA1 can induce MMP-9 (50) which can enzymatically cleave SAA1 (Figures 1 and 2). However, the biological consequences of SAA1 processing are poorly studied (31, 32, 51). Several functions have been ascribed to SAA, of which chemotaxis of leukocytes, induction of chemokines and matrix degrading enzymes reach the highest specific activity (3, 17, 29, 50, 52–55). Moreover, both MMPs (e.g. MMP-9) and chemokines (e.g. CXCL8 and CCL3) are co-induced in monocytes by SAA1 (17, 50). Furthermore, truncated CXCL8 can synergize with SAA1 in leukocyte chemotaxis (17, 21, 49).

Matrix metalloproteinase-1, MMP-2, and MMP-3 cleave SAA, with cleavage sites between residue 57 and 58 for MMP-1, between 51 and 52 for MMP-2, and between residues 56 and 57 and 57 and 58 for MMP-3. In addition, MMP-2 generates the NH_2_-terminal fragment SAA1(1–51) (31, 32, 51). Here, we demonstrate that MMP-9 was found to rapidly (within 30 min) cleave human SAA1 yielding a significant conversion after 3 h into the COOH-terminal fragments SAA1(58–104), SAA1(52–104), and SAA1(57–104). These fragments represent 50, 30, and 20% of the total amount cleaved SAA1, respectively. Since none of these fragments had been biologically characterized, SAA1(58–104), SAA1(52–104), and the complementary NH_2_-terminal peptide SAA1(1–51) were chemically synthesized and purified to homogeneous peptides with *M*_r_ of 5,256.2, 5,884.6, and 5,815.7, respectively. Although SAA1 is a potent chemokine inducer in monocytes *via* TLR2 (21) [or other receptors, such as TLR4 (23)], the COOH- and NH_2_-terminal peptides failed to induce CXCL8 in these cells, indicative for loss of TLR2 signaling. Functional TLR2 expression on fibroblasts and CD14^+^ monocytes has been described (56), and peptidoglycan triggered CXCL8 production in the cells tested here (data not shown). However, the involvement of other receptors, such as TLR4, is not excluded (23). Unlike intact SAA1, the peptides SAA1(1–51), SAA1(52–104), and SAA1(58–104) did not chemoattract neutrophils. In contrast to SAA1(1–51), the COOH-terminal fragment SAA1(58–104) was nevertheless still able to cooperate with CXCL8 in neutrophil activation and chemotaxis assays. This effect could be blocked by a selective FPR2 antagonist indicating that the GPCR-binding capacity of SAA1(58–104) was partly preserved. In addition to the blockage of SAA1(58–104) by a specific FPR2 antagonist, the use of FPR2 by an SAA1 fragment has been evidenced by its capacity to prevent the chemotactic response of neutrophils to intact SAA1. Indeed, SAA1(47–104) desensitized the chemotactic response of neutrophils toward cooperating intact SAA1 and CXCL8 (34). However, in binding competition experiments on neutrophils and FPR2-transfected HEK293 cells with NH_2_-terminally TAMRA-labeled MMK-1, SAA1(58–104) could not displace the specific and strong FPR2 ligand MMK-1 (data not shown). Moreover, pretreatment of FPR2-transfected HEK293 cells with 1 µg/ml intact SAA did not desensitize the calcium response to 10 ng/ml of the FPR2 agonist WKYMVm (data not shown). Thus, we can only provide indirect evidence that SAA1 fragments still bind to FPR2, and we do not exclude that other receptors are also involved. Similar to SAA1(58–104), SAA1(47–104) failed to directly chemoattract neutrophils. This fragment also lacked monocyte chemotactic activity and the capacity to induce chemokines. By contrast, SAA1(47–104) synergized with CCL3 to induce monocyte migration (34). In addition, Zhou et al. demonstrated that SAA1(11–58), like SAA1(1–51) was unable to activate FPR2 (57). Hence, we can conclude that for the most important biological activities ascribed to SAA1, in particular chemokine induction and direct chemotactic activity, the intact structure of SAA1 needs to be preserved. However, COOH-terminal fragments, but not NH_2_-terminal fragments, retain their capacity to cooperate with chemokines in chemotaxis.

Little is known about the role of MMP-9-generated COOH-terminal fragments of SAA1 in pathology, whereas the literature on NH_2_-terminal fragments is more comprehensive (29, 30, 32, 58–60). In particular MMPs are detected during amyloidosis in amyloid A (AA) deposits together with NH_2_-terminal fragments of SAA1 (51). Amyloidosis secondary to chronic inflammatory diseases, such as rheumatoid arthritis, is caused by the systemic deposition of insoluble AA fibrils in various organs (29, 32). SAA is considered as the precursor of AA fibril protein deposited during this disease (29, 50, 61, 62). The amyloid fibrils found in patients with AA amyloidosis largely consist of SAA(1–76), as the predominant AA protein, although NH_2_-terminal fragments of different lengths have been reported (31, 51, 58, 63–65). However, the NH_2_-terminal SAA1(1–51) fragment, investigated in this paper, has not been described in the literature as part of AA amyloid deposits.

In conclusion, we demonstrated that the inflammation-associated MMP-9 generates SAA1 COOH-terminal fragments with impaired TLR2-mediated chemokine-inducing capacity, while retaining their FPR2-mediated potential to cooperate with chemokines in leukocyte activation and attraction. The TLR2-mediated cytokine-inducing capacity of SAA1 assists in initiating an inflammatory response, but later on in this process, when MMPs are released, the inflammatory capacity of SAA1 is fine-tuned and only the cooperative interactions with chemokines remain to prolong the duration of inflammation.

## Ethics Statement

This study was carried out in accordance with the study protocol (S58418) that was approved by the ethical committee of the KU Leuven with written informed consent from all subjects. All subjects gave written informed consent in accordance with the Declaration of Helsinki.

## Author Contributions

MG: planned and performed experiments, analyzed data, wrote part of the manuscript and submitted the manuscript; MDB: planned and performed experiments, analyzed data, wrote part of the manuscript; SAS: performed experiments, analyzed data and corrected the manuscript; JV: was involved in proteolytic cleavage of SAA; SK, NP, LV and NB: performed experiments; GO: was involved in proteolytic cleavage of SAA and corrected the manuscript; PP: executed biochemical quality control of reagents, performed protein synthesis and corrected the manuscript; JVD: analyzed data, designed the study, corrected the manuscript; SS: designed the study, gave technical advice, analyzed data, corrected the manuscript.

## Conflict of Interest Statement

The authors declare that the research was conducted in the absence of any commercial or financial relationships that could be construed as a potential conflict of interest.
